# Evolution of Spiral and Scroll Waves of Excitation in a Mathematical Model of Ischaemic Border Zone

**DOI:** 10.1371/journal.pone.0024388

**Published:** 2011-09-15

**Authors:** Vadim N. Biktashev, Irina V. Biktasheva, Narine A. Sarvazyan

**Affiliations:** 1 Department of Mathematical Sciences, University of Liverpool, Liverpool, United Kingdom; 2 Department of Computer Science, University of Liverpool, Liverpool, United Kingdom; 3 Pharmacology and Physiology Department, The George Washington University, Washington D. C., United States of America; Georgia State University, United States of America

## Abstract

Abnormal electrical activity from the boundaries of ischemic cardiac tissue is recognized as one of the major causes in generation of ischemia-reperfusion arrhythmias. Here we present theoretical analysis of the waves of electrical activity that can rise on the boundary of cardiac cell network upon its recovery from ischaemia-like conditions. The main factors included in our analysis are macroscopic gradients of the cell-to-cell coupling and cell excitability and microscopic heterogeneity of individual cells. The interplay between these factors allows one to explain how spirals form, drift together with the moving boundary, get transiently pinned to local inhomogeneities, and finally penetrate into the bulk of the well-coupled tissue where they reach macroscopic scale. The asymptotic theory of the drift of spiral and scroll waves based on response functions provides explanation of the drifts involved in this mechanism, with the exception of effects due to the discreteness of cardiac tissue. In particular, this asymptotic theory allows an extrapolation of 2D events into 3D, which has shown that cells within the border zone can give rise to 3D analogues of spirals, the scroll waves. When and if such scroll waves escape into a better coupled tissue, they are likely to collapse due to the positive filament tension. However, our simulations have shown that such collapse of newly generated scrolls is not inevitable and that under certain conditions filament tension becomes negative, leading to scroll filaments to expand and multiply leading to a fibrillation-like state within small areas of cardiac tissue.

## Introduction

Heart is a remarkably reliable machine whose function is to pump the blood as required by the organism. An important part of its work is the orderly propagation of electrical signal, that is the wave of excitation passing through cardiac muscle, which subsequently triggers its ordered contraction. Abnormalities of the excitation wave propagation, known as arrhythmias, are precursors of sudden cardiac arrest and other life-threatening pathologies. This paper focuses on mathematical analysis of arrhythmogenic conditions associated with cardiac tissue recovery from acute ischemia, also known as *reperfusion arrhythmias*. Such recovery can be more dangerous then ischemia itself and often leads to ventricular fibrillation and sudden cardiac death [1]. Reperfusion can be spontaneous (relief of coronary spasm, dislodging of a thrombus) or externally imposed (antithrombolitic therapy, angioplasty). It can also occur on a microscopic scale during ischemia itself as a result in shifts in microcirculation [2]. As of today, the exact mechanisms of reperfusion arrhythmias remain poorly understood. This is because the inner layers of ischaemic boundary are inaccessible for live visualization on a spatial scale required to distinguish behaviour of individual cells. Therefore, in order to understand how the abnormal activity spreads from single cells to the bulk of cardiac tissue, we and others had to rely on either *in vitro* experimental preparations or on computer modeling.

Our work builds on the experimental data acquired from monolayers of cardiac myocytes under conditions that mimicked the ischaemic boundary [3]–[5], and the results of direct numerical simulations that closely matched these experimental observations [5]–[7]. The *in silico* modelling provided an explanation to several experimental findings, including the dependence of drift of boundary-bound spirals on their chirality, pin-drift-pin type of spiral tip motion and the effect of boundary movement on spiral detachment [6], [7].

**Figure 1:**
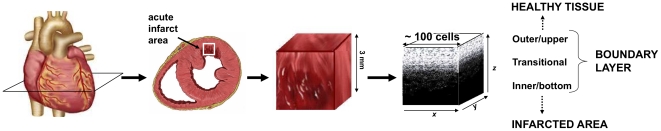
We consider excitation dynamics on a microscopic spatial scale, in areas of cardiac tissue with severely suppressed cell-to-cell coupling superimposed with elevated cell excitability.

The rotating waves of activity to be discussed in this paper, occur on a much smaller spatial scale as compared to classical cardiac reentry [8]–[12], see figure 1. Specifically, we are focusing on a dynamically and spatially changing set of conditions which can occur within a thin layer of cells sandwiched between intact healthy tissue and the recovering ischaemic areas. Myocytes within such layers can become spontaneously active as a result of calcium overload and/or local noradrenaline release. The impact of intrinsic myocyte heterogeneity on network behaviour is markedly enhanced due to decrease in electrical coupling between the cells. It gets even more complicated as the physicochemical factors that create the boundary, such as low pH, lack of oxygen, hyperkalemia, noradrenaline, move in space due to the dynamic nature of reperfusion. Altogether the moving boundary, heterogeneous substrate, steep gradient of coupling and self-oscillatory activity of individual cells can give rise to a rich network behaviour discussed in our previous paper [7]. A continuous generation of mini-reentries from individual ectopic sources occurs within the least coupled cells layers, and then the activity spreads towards the better coupled layers of the boundary [8]. This scenario was suggested by our experiments in neonatal rat cardiomyocytes and was later supported and expanded upon using the *in silico* approach. Yet, numerical modelling of cellular behaviour has its limitations, and there is a need to understand how much of the phenomena observed in the simulations are generic and how much of it depends on the specifics of the model. Further still, cardiac tissue is three-dimensional, whereas our experiments and simulations reported previously were conducted using two-dimensional cell networks. Extrapolation of the two dimensional data into three dimensions requires additional theoretical understanding.

In the present paper, we use an asymptotic theory of spiral and scroll waves' drift together with the recently developed numerical technique to compute the response functions of spiral waves [13]–[15] to provide theoretical analysis of our experimental and numerical data. We then use this theoretical framework to predict behaviour of the scroll waves in an ischaemic border zone in 3D, where such experiments are not currently feasible. Finally we confirm theoretical 3D predictions by numerical simulations of cell network behaviour.

Specifically, we address the following questions:

1. In both experiments and numerical simulations, spiral waves were not static within the border zone. What determines the components of the drift velocity, and why the spiral cores can be dragged together with the moving border zone?
2. In both experiments and numerical simulations, the drift of the spirals was interrupted by their “pinning” to clusters of cells. We have shown numerically that these can be cell clusters of either elevated or suppressed excitability. What is the mechanism of such pinning?
3. In both experiments and numerical simulations, the episodes of spiral drift and pinning alternated. What is the mechanism by which pinning can give way to further drift?
4. One of arrhythmogenic scenarios proposed in [6], [7] involved pinning of a spiral wave to a local heterogeneity which persists long enough until the border zone passes and the spiral gets into the better coupled tissue. Is this scenario viable in 3D?

## Methods

### Direct Numerical simulations: tissue model

**Figure 2:**
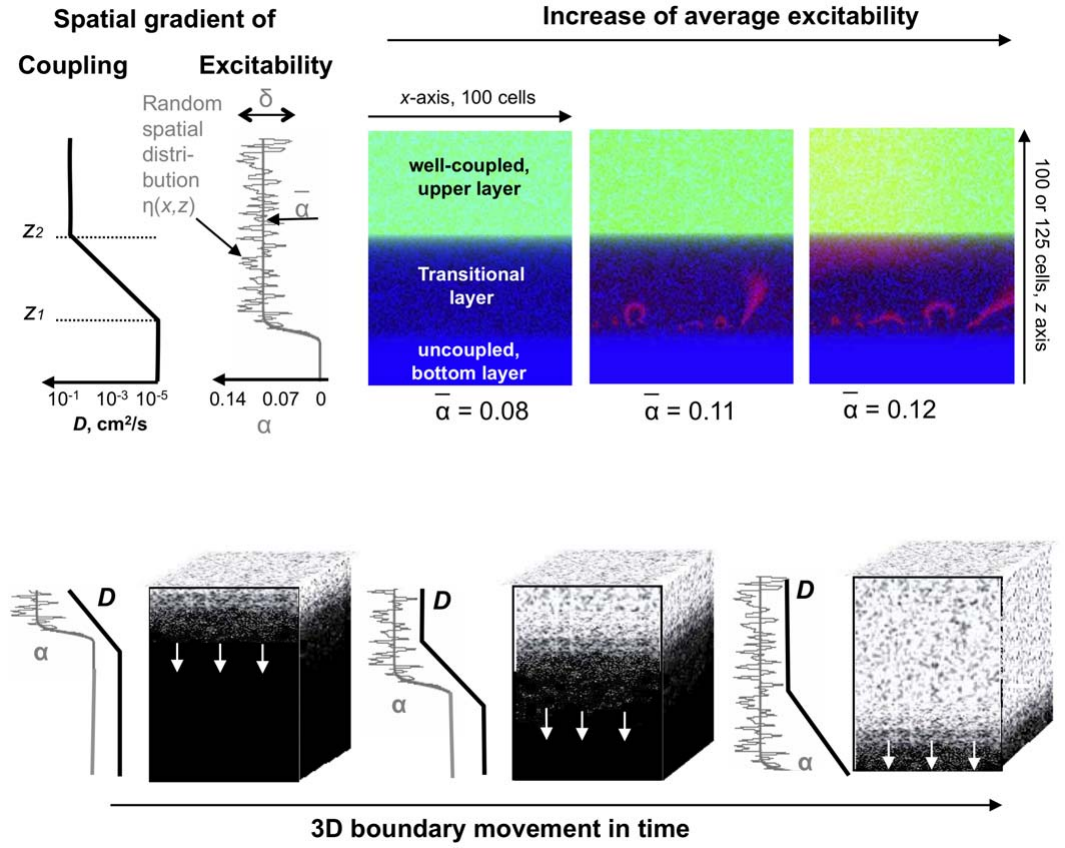
Schematic of numerical protocols. Top row: 2D setting [7]. Distribution of the diffusivity 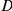 and excitability/automaticity 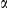 across the border zone. The three colour panels are representative snapshots of solutions at different values of 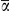, as it was slowly growing at a fixed profile of 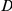. Here and below we use the red colour component to show the excitation wave (transmembrane voltage), blue component for the cell excitability/automaticity (denoted as 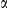, see definition in the text) and the green component for the cell electrical coupling strength (denoted as 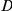 for transmembrane voltage diffusivity). E.g. yellow is a sum of green and red, and magenta is a sum of red and blue. Bottom row: 3D setting for this paper. The transition zone moves downwards.

**Figure 3:**
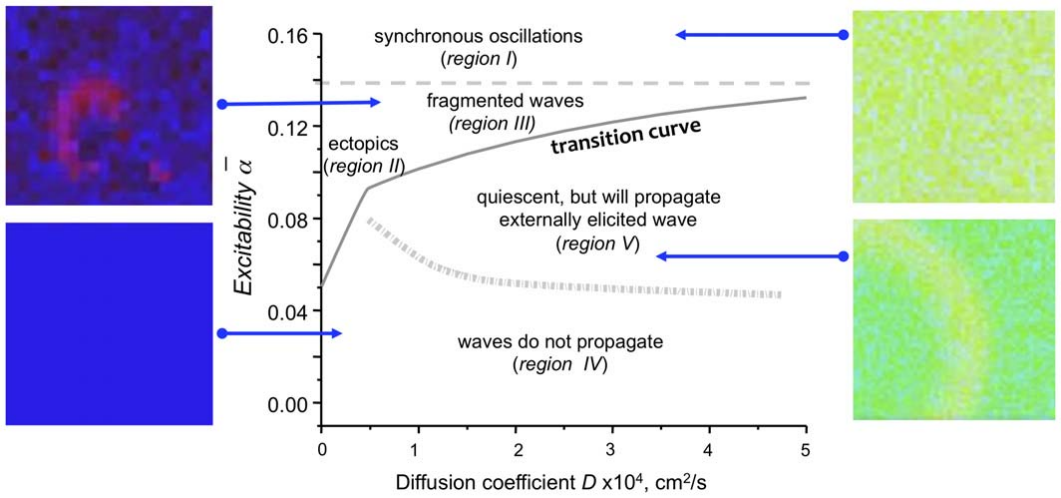
The parameter space diagram of the numerical model (1, 2) heterogeneity) [**6**]. The parameter regions I-V correspond to distinctive regimes of wave initiation and propagation, observed in simulations where 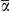 and 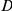 were maintaned constant throughout the simulations. The panels on the sides show representative snapshots of solutions, corresponding to regions I, III, IV and V.

The mathematical model mimicking the conditions when tissue recoveres from acute ischaemia, and its experimental foundations are described in detail in our previous works [7] and references therein. To capture the complexity of pathophysiological conditions associated with reperfusion arrhythmias, we use a simplified kinetic model of individual cells, and enrich it by adding individual cell heterogeneity, different course of recovery of cell coupling and excitability, and spatial arrangement of conditions on the boundary of ischemic tissue. The importance of the latter three factors, cell heterogeneity, individual cell excitability and cell-to-cell coupling, for the cardiac network behaviour was studied in our previous paper [6]. The arguments and experimental evidence presented there suggest that from the network/tissue perspective, it is not very important exactly how these properties are altered. In this paper, we model a tissue recovering from acute ischaemia as a three-layered slab made of a heterogeneous mix of cells, subject to a vertical gradient of average cell excitability and a vertical gradient of cell-to-cell coupling strength. The 2D and 3D versions of the model are illustrated in figure 2. In terms of the parametric diagram described in [6] and shown in figure 3, the bottom layer corresponds to the parametric region IV. It has low excitability and weak coupling which result in the quiescent state where propagation is not possible. The outer layer with high excitability and strong coupling is in the parametric region V of the digram, corresponding to the quiescent state where wave propagation is possible. The middle, or transitional, layer is sandwiched between inner and outer layers, so, from bottom to top, it starts in region III (high excitability and weak coupling resulting in spontaneous fragmented waves) and then via a gradual increase in coupling strength proceeds to region V (high excitability, strong coupling, quiescent state where wave propagation is possible) characteristic of the upper layer. The layers are not static but move downwards through the slab, which represents the reperfusion, or wash-out, of the agents affecting the relevant tissue properties. Depending on type of reperfusion, blood flow can recover within seconds (cases of resolved coronary spasm, spontaneous dislodging of thrombi, angioplasty) or within minutes (cases of changes in coronary flow due to gradual accumulation of metabolites or pharmacological interventions). Therefore, the dynamics of moving border zone can vary in a rather wide range, from 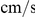 to 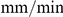. We select the values of the border zone speed that produce interesting effects.

We assume that the cells are arranged in a rectangular grid of 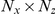 (in 2D) or 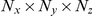 (in 3D) cells connected to each other via Ohmic resistances. Properties of the cells and resistivities of the contacts are varied in time an space. The cells are assumed to have linear size of 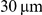 which serves as a space scale to endow the voltage diffusivity and other space-related quantities with suitable dimensionality. The cells are connected to the nearest neighbours, so an internal cell has four contacts in 2D and six contacts in 3D.

The excitable dynamics of cells is described by the Beeler-Reuter-Pumir [6] (BRP) model of a neonatal cardiac myocyte. The BRP model is based on the generic Beeler-Reuter [16] model of a cardiac myocyte, which contains an explicit, albeit simplified, description of individual ionic currents, and was slightly modified to match the ionic currents reported for neonatal cardiac cells used in our experiments [6]. The complete set of the BRP model equations is given the Appendix S1; here we only outline the modifications. The affected equations are
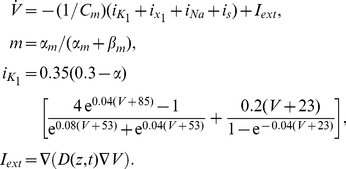
(1)The last equation in (1) is written, for brevity, as the continuous limit, whereas actual calculations of the inter-cellular currents were discrete, as described in more details in the Appendix S1. The coupling strength between the cells is represented by the voltage diffusion coefficient 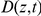, and some of the values of 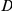 we use here are too low to hold the continuous limit of the (1). Note that as far as the continuous limit is concerned, the voltage diffusivity 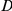 is the only quantity in the model related to space, so while within this limit, all results are easily rescaled from one value of 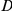 to another.

The maximum permeability of the fast inward current 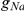 is 60% of the standard (2.4 vs 4), and that of the slow inward current, 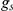, is 50% of the standard (0.045 vs 0.09).

We also have altered the balance between inward and outward currents by inhibiting the inward potassium rectifier current, 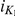 [6], [17], [18]. Suppression of 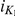 to 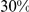 of the standard value mimics its smaller contribution reported for neonatal cardiomyocytes [19], [20] as compared to the original Beeler and Reuter values for adult ventricular cells [16]. We use this supressed value of 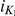 (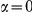) for the bottom layer of the ischemic slab. In the upper layers, further suppression, represented by the factor 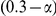, 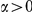, enhances excitability. For high enough values of 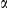, this leads to spontaneous firing of individual cells, *i.e.* makes them automatic [6]. In [6] we considered 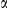 values that led to the *in silico* network behaviour closely matching the behaviour of neonatal cardiomyocyte layers. The excitability of the latter cells was increased using beta-adrenergic stimulation with isoproterenol [5] and ischaemia-reperfusion protocol [4]. Compared to [6], here we only consider a narrow range of values of 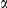, where phenomena interesting for our present study are observed. In our previous paper [7], parameter 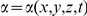 varied in space and time and it was essential that it covered both excitable and automatic regimes, so it was called both “automaticity” and “excitability”. Here we concentrate mostly on the events happening in the excitable regime (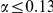, within the range of intermediate coupling values, or region V in the parametric space, shown in figure 3), hence for brevity we mostly refer to parameter 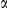 as “excitability parameter” or simply “excitability”. It should be kept in mind, however, that due to the above ambiguity, this usage may differ from the meaning of “excitability parameter” in other studies.

Heterogeneity of individual cells' excitability is described as
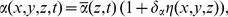
(2)where 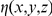 is the Gaussian distributed uncorrelated random variable with unit dispersion, and parameter 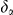 represents the intensity of heterogeneity.

Space-time variations of 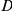 and 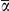 are defined as
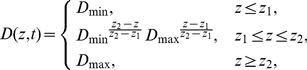
(3)and
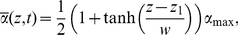
(4)where 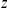 is the coordinate across the boundary, 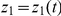 and 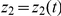 are the limits of the steepest part of the coupling gradient, 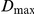 is the diffusion coefficient in the upper, well-coupled layer, 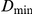 corresponds to the bottom, uncoupled layer, and 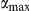 is the highest level of excitability within the slab. We used the boundary width 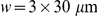 in all simulations. Parameters 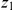, 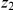 vary linearly in time, 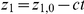, 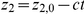.

Thus, the recovering ischaemic tissue is modelled as layers with imposed excitability and coupling profiles as shown in figure 2. Specifically, we are modelling experimental conditions when previously severely uncoupled ischaemic areas are reperfused with agents which elevate cell excitability.

Finally, we also made simulations with deliberately arranged parametric distributions not exploiting random number generators. The details of those are given where the results are described.

### Asymptotic theory of drift

The asymptotic theory of spiral and scroll dynamics under small perturbations [13], [15], [21]–[23] is formulated for the “reaction-diffusion” system of partial differential equations (PDEs),
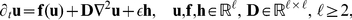
(5)where 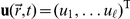 is a column-vector of the reagent concentrations, 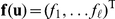 is a column-vector of the reaction rates, 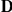 is the matrix of diffusion coefficients, 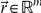 (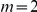 or 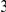) is the vector of coordinates, and 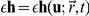 is some small perturbation of the right-hand side, 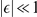. For the Beeler-Reuter-Pumir model, 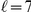, and 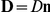, where 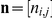, 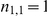 and 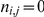 otherwise.

The theory assumes that spiral wave solutions to equations (5) for 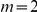 are stationary rotating, not meandering. This is indeed satisfied for BRP model for all 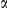 values considered. Mathematically, the assumption means that a spiral wave solution to (5) for 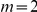 in the 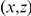-plane has particular dependence on space and time, so it rotates around a center of rotation 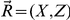 with angular velocity 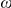 and fiducial phase 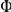


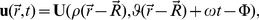
(6)where 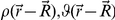 are polar coordinates centered at 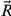. A spiral wave can of course rotate in either direction; we assume 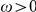 for clockwise rotation.

In presence of a small perturbation, 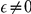, a spiral wave preserves the pattern, only slowly changing its frequency and location of the core. It actually behaves as a localised object, only sensitive to perturbations affecting its core. The localised sensitivity to perturbations is mathematically expressed in terms of the spiral wave's response functions, that is the critical eigenfunctions of the adjoint linearised operator, which are essentially nonzero only in the vicinity of the core and exponentially decay with distance from it. Knowledge of the response functions allows quantitatively accurate prediction of spiral waves drift due to small perturbations of any nature, which makes the response functions a property that is as fundamental for spiral waves as mass is for matter. In particular, the 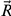-drift velocity, *i.e.* the velocity of the drift of the position of the core of the spiral, is defined, in the first order in 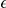, by an integral of the perturbation 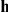,
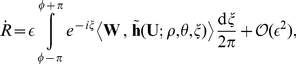
(7)where 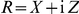 is the complex coordinate of the instant spiral centre, inner product 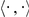 stands for the scalar product in functional space,
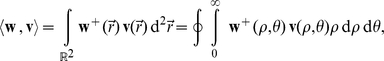
function 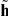 is perturbation 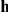 of the right-hand side in (5) , re-written in the 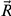-centered corotating frame of reference 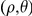, where 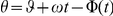 is the polar angle in the corotating frame of reference, and 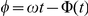 is the time measured in terms of the spiral rotational phase. The kernel 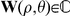 of this integral is the (translational) response function which characterizes the unperturbed spiral wave solution (6) and can be calculated numerically together with it. Given the dependence of the perturbation 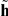 on the current position of the spiral 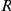, equation (7) is a closed system of ordinary differential equations (ODEs) for the coordinates of the instant centre of rotation of the spiral wave.

**Figure 4:**
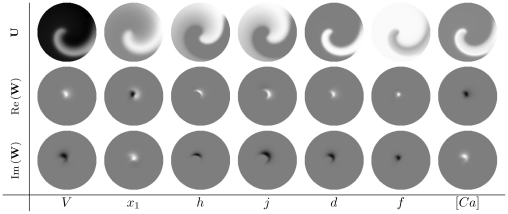
Density plots of the components of a spiral wave solution 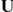 and its translational response function 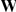. Parameter 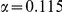. The radius of the disk is 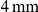 assuming 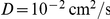. In each plot, white corresponds to a value 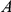 and black corresponds to 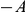 where 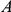 is chosen individually for each plot, *e.g.* for the 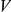-component of 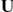, 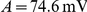. The grey periphery of the 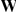 plots, the second and third rows, corresponds to 0.

A more detailed exposition of the theory and description of the method of calculating the response functions are given in [14], [15]. In the present study we use the same method with the modifications relevant to the BRP model, which has 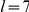 as opposed to simplified 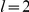 models considered in [14], [15]. Figure 4 shows density plots for the spiral wave, 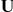, and its response functions, 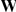, in BRP model for 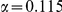; for other values of 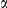 the plots look qualitatively similar. The important property is that all components of the response functions are large only in the core of the spiral and quickly decay beyond it.

Scroll waves are three-dimensional analogues of spiral waves. They rotate around curves called *filaments*, as spiral waves rotate around points called centres. In general, scroll filaments are not fixed in space but move, typically on a slow timescale relative to the rotation period. Hence, in addition to whatever dynamics 2D spiral waves might have, scroll waves exhibit additional dynamics associated with filament motion [24]–[31]. Working in Frenet coordinates, the motion may be conveniently expressed in terms of the velocities 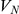 and 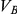 in the normal and binormal directions, respectively, at each point along the filament. Motion along the tangential direction is of no physical significance and is equivalent to reparametrization of the filament.

Then, the motion equation for the filament, in the assumption of small filament curvature, 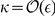, and slowly varying phase, has the form [21], [32]–[34]

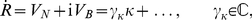
(8)where omitted are terms representing effects of the perturbations of the right-hand sides, if any (which may be of the same order as that shown), and higher-order terms. The complex coefficient 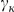 in the equation (8) is calculated using the same response functions as for the underlying spiral wave, as
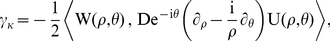
(9)and the positive sign of 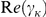 means movement towards the local centre of curvature.

Following [32], some publications use the notation 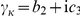. As shown in [21], [33], the real component 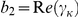 has special importance: if 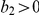, the overall length of the filament becomes shorter with time, and if 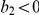, the filament lengthens with time, as long as the asymptotic description remains valid. Hence this coefficient is sometimes called *filament tension* of the scroll wave. The coefficient 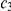 is the *binormal drift coefficient* and describes the drift of a scroll ring filament perpendicular to the plane of the ring, or more generally, the velocity component orthogonal to the local plane of the filament.

#### Superposition principle

Since the right-hand side of (7) is linear in 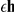, the 1st-order asymptotic theory obeys a superposition principle: if the overall perturbation is a sum of several components,
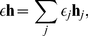
(10)then the overall drift velocity is determined by the sum of the corresponding partial “forces”,
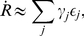
(11)where 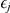 is the magnitude of the 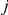-th perturbation, and 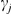 is the force produced by a unit perturbation of that sort, hereafter referred to as “specific force”, given by
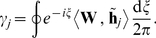
(12)In the setup of our present study, the forces acting on a spiral or scroll wave of excitation within the recovering ischaemic tissue are caused by the filament curvature (described by specific force 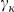), the localised inhomogeneities and the smooth gradient of parameter 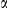 (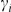 and 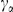 respectively), and the gradient of diffusivity (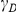). We shall now present the explicit form of the the relevant perturbations and the forces.

#### 2D curvature drift

It had been shown [25] that due to the axial symmetry of a scroll ring solution, there is a strong connection between the scroll ring filament's motion in 3D and drift of the core of a spiral wave in response to applied electric field (electrophoretic drift) in 2D. For the corresponding perturbed 2D reaction-diffusion equation,
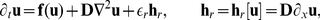
(13)the specific force 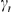 of the electrophoretic drift is given by [15], [23]

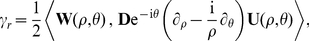
(14)which is exactly the opposite of 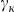 given by (9) . The opposite sign can be understood if one remembers that the positive sign of 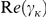 means movement towards the local centre of curvature of the filament, and the form of the perturbation (13) with positive 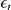 corresponds to the centre of curvature located at the line 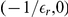, *i.e.* in the negative 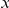 direction with respect to the current spiral centre [25].

This equivalence of 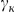 and 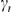 up to the sign allows us to use the 2D simulations of system (13) to estimate the drift velocity of a 3D scroll ring, and hence estimate the 3D coefficient 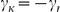. Subsequently, these 2D estimations can be used to verify/confirm both drift velocities 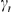 and 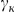 obtained using the response functions in (14) and (9).

#### Smooth gradient of excitability

We suppose that the excitability kinetic parameter 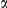 varies in space,
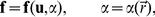
(15)and, further, that the profile 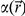 is smooth enough and can be approximated by a linear spatial gradient, within the spiral core where the components of the response functions are essentially non-zero,
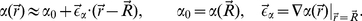
(16)Then, the velocity of the drift induced by the parameter 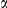 gradient works out [15] as
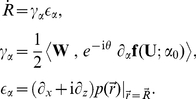
(17)The real part of 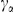 gives the component of the drift velocity along the gradient of 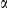 and is positive if the drift is towards higher values of 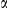. The imaginary part of 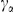 describes the drift across the gradient of 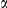; it is positive if the lateral component of the drift velocity is counter-clockwise with respect to the direction of 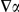.

#### Localized inhomogeneity of excitability

As can be seen from figure 4, the core size of the spiral wave in BRP model is 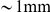 for 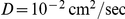. A 1000-fold decrease of 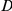 down to 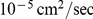 implies shrinkage of the core to the size of one cell, 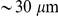. Hence for the coupling values at the lower end of the range, localized heterogeneities of 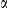 become of principal importance, and they cannot be considered as smooth gradients.

To elucidate possible role of the localised inhomogeneities, let us consider the case when the continuous limit is still applicable, but the spiral core size is comparable with the size of a localized inhomogeneity, or the magnitude of such inhomogeneity is so significant it affects the spiral dynamics despite the small geometry size. This can happen when the random distribution of properties produces relatively large lumps of cells with local average excitability deviating from the overall average. Let's consider an idealized situation when the parametric inhomogeneity is localized in a disk of radius 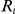 centered at 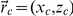 and is uniform within it, so
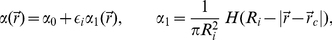
(18)where 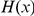 is the Heaviside step function. Then for a small enough 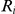, the velocity of the drift induced by the localized inhomogeneity is defined as [15], [35]

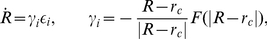
(19)where 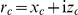 and
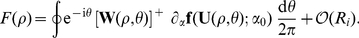
(20)Here 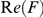 is the radial component of the drift velocity, positive if the spiral moves towards the centre of the inhomogeneity, and 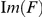 is its azimuthal component, positive if clockwise with respect to the centre of inhomogeneity.

#### Gradient of the diffusivity

**Figure 5:**
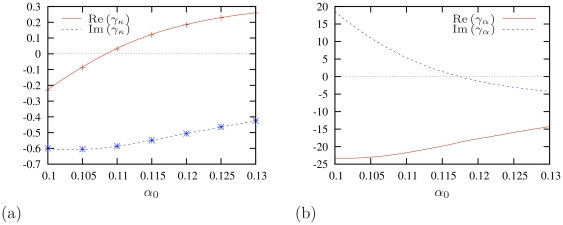
Dependence of the specific forces 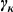 and 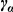 on the unperturbed value of the excitability parameter 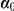. Diffusion coefficient 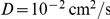. (a) Specific force 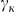 caused by filament curvature 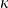. Note that 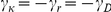. Symbols “+” and “
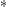
” show estimates of 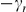 from direct numerical simulations of (13) , for comparison. The difference between predicted and simulation values is smaller than 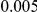 at all points. (b) Specific force 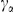 caused by the gradient of excitability parameter 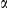. Red solid lines: real parts, the longitudinal components. Dashed blue lines: imaginary parts, the lateral components. The meanings of the vertical axes are different for different curves and are designated in the legends.

We also deal with the drift caused by a gradient of the diffusivity, so that
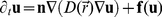
(21)Suppose the diffusivity varies smoothly, so it can be approximated by a linear function within the core of the spiral,
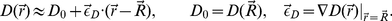
(22)Substituting this into (21) , we get the perturbed reaction-diffusion equation of the form (5) with 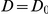 and the perturbation
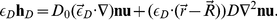
(23)This leads to the expression for the specific force induced by the gradient of the diffusivity in the form
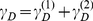
(24)where
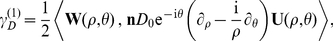
(25)and
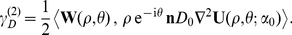
(26)It is easy to see that the specific force 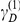 in (25) coincides with the 2D electrophoretic drift specific force 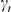 given by (14) up to the substution 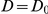. On the other hand, Dierckx [34] has shown that the problem of drift in the gradient of diffusivity is equivalent to the problem of 2D electrophoretic drift, up to a transformation of coordinates. This implies that 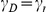, and since 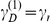, the integral (26) should be zero. In our calculations using response functions, the values of 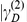 do not exceed 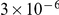 for the whole range of 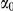 considered, which is indeed small compared with typical values of 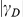 shown in figure 5 (a) (note that 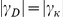). This small deviation of the calculated value of 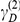 from zero serves as a measure of accuracy of the response function and the integrals based on it. Note that specific forces correspond to the limit of 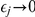. Direct numerical simulations presented in [36], performed at finite values of 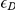 and in a different model, show empirical values of 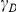 and 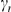 differing by as much as 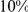.

## Results

### Continuous limit: predictions from the asymptotic theory

#### Effects of elementary perturbations

Based on the response functions shown in Figure 4 , we have computed the values of the specific forces acting on spiral (2D) and scroll (3D) waves under conditions associated with recovering ischaemic border. These forces include the specific force 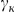 caused by the curvature of the vortex filament, the specific force 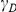 caused by the gradient of diffusivity, the specific force 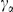 caused by the gradient of parameter 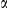 and the specific force 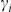 caused by a localised inhomogeneity of parameter 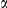.

Figure 5 (a) shows the theoretical predictions for the components of the specific force 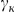 caused by the curvature of the vortex filament. That panel also shows an excellent agreement of these predictions with the results of the direct numerical simulations of electrophoretic drift (13) (remember that 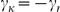). The components of 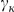 correspond to the two filament's drift coefficients: the “filament tension” 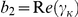, and the *binormal drift coefficient* 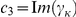. The *binormal drift coefficient* 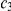 determines *e.g.* the drift of scroll rings along their axis. The filament tension 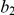 is usually much more important for a scroll's dynamic, as the positive filament tension means that the filament will tend to straighten or collapse if geometry allows it. Negative filament tension means that the filament will tend to spontaneously lengthen and curve and can produce “scroll wave turbulence” which is phenomenologically similar to fibrillation [21], [25], [33], [37], [38]. An important observation from Figure 5 (a) is that in the shown interval of parameter 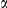, filament tension 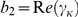 changes the sign and is overall smaller than the binormal drift coefficient 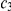.

As discussed above, the drift caused by the curvature of the filament is equivalent to the drift caused by the gradient of the diffusion coefficient, so the same coefficients, though taken with the opposite sign, will describe the drift of the spiral core or scroll filament in response to gradient of diffusivity. Namely, coefficient 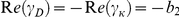 will determine the component of the drift along the gradient of diffusivity and 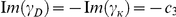 across it. Following figure 5 (a), 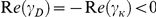 at higher values of 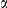, and 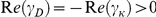 at lower values of 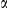. So, at higher values of 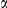 the negative specific force of the gradient of diffusivity will drag the spirals towards poor coupled regions with smaller diffusion, while at lower values of 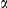 the positive specific force of the gradient of diffusivity will drag the spirals towards better coupled regions with higher diffusion. Thus, the fact that 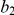 changes sign in the relevant range of parameters, means that the diffusivity gradient can either drag spirals towards the pourly coupled bottom layer or repell them into the better coupled upper layer, depending on the local value of excitability parameter 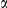. Also, the fact that 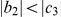 means that the spirals should move preferentially across the diffusivity gradient that is along the border zone, which agrees with the numerics and experiments.

Figure 5 (b) shows the theoretical predictions for the drift coefficients in response to a smooth gradient of parameter 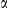. Here, an important feature is that the longitudinal coefficient 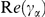 is negative in the whole range of 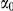. This means that the spirals should drift towards areas with lower excitability. This agrees with the general rule noted *e.g.* in [39], [40].

**Figure 6:**
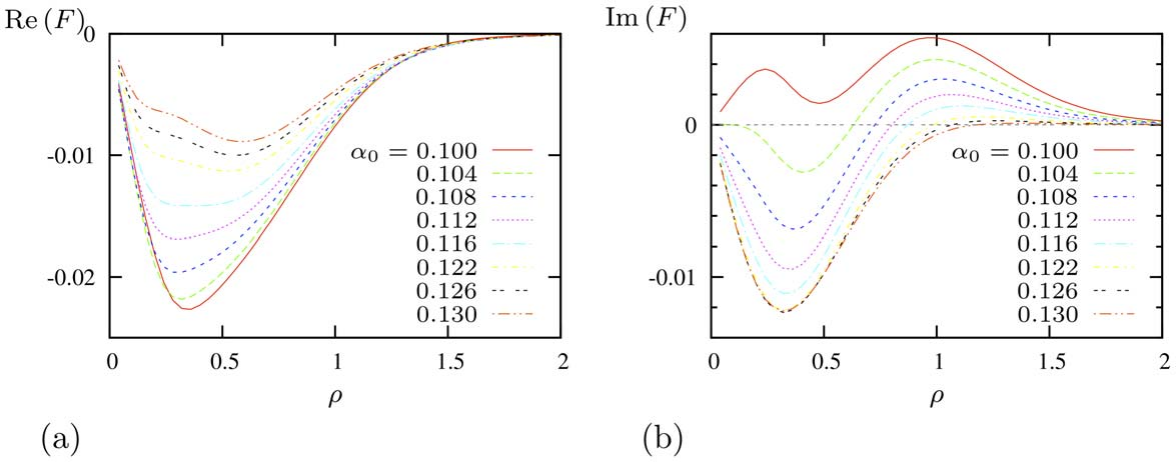
Interaction with point-like inhomogeneity. Dependences of (a) radial and (b) tangential components of the specific force caused by point-like inhomogeneity of excitability 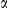, on the distance from instant spiral rotation centre to the inhomogeneity, at selected values of background excitability 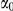 as indicated in the legends. The scale of 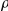 is given in 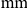 assuming 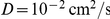.

Figure 6 shows the theoretical prediction for interaction of a spiral wave with a point-like heterogeneity in parameter 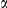. Here, the interaction force depends on the distance between the spiral's centre and the heterogeneity. The negative sign of the radial component 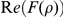, observed for all distances 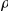 and all values of 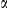 considered, means that a localized inhomogeneity with lowered excitability, 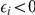, should attract spiral waves, and those with higher than the background excitability, 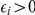 should repel them. This is also intuitively consistent with the predictions for the linear gradient of 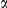 given by figure 5 (b).

The constant sign of the inhomogeneity specific force 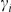 radial component 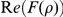 in figure 6 (a) is not a general case, and in other models the sign of interaction with a localized inhomogeneity may depend on the distance to it, which may lead to “orbital” motion around such inhomogeneity, with orbit radii at the zeros of 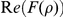 [35]. So, following the graphs in figure 6 (a) and the shown constant sign of the radial component 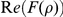, we should not observe an orbital motion in our present BRP model.

#### Complex perturbations and pinning/unpinning in 2D

We shall now use the superposition principle to analyse the 2D drift of a spiral wave subject to a combination of forces caused by a smooth gradient of diffusivity, a smooth gradient of the excitability parameter 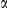 and a localised inhomogeneity in parameter 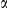. In a system of reference with the origin at the centre of the disk inhomogeneity, 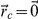, the equation of motion for a complex coordinate of spiral wave rotation centre 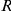 is
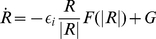
(27)where 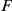 is the force induced by the localised inhomogeneity and 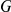 is the constant dragging force due to the smooth gradient of parameter 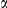 and/or the diffusivity gradient. We use polar coordinates for the instant centre position, 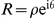, and also set 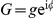 where 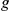 and 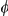 are the magnitude and direction of the gradient force. Further, we separate the radial and azimuthal components of force 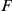, 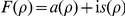. Then, the equations of motion in the two real dynamic variables are
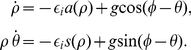
(28)An equilibrium in the system (28) may be observed at a radius 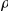 satisfying
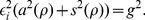
(29)It is easy to see that equilibria will not exist, that is, the smooth gradient force will definitely tear a spiral off from the localized inhomogeneity, if
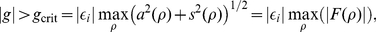
(30)that is, if the gradient force exceeds the maximal force of interaction with inhomogeneity, including both radial and azimuthal components (see also [41] where a special case with 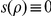 was considered).

Following (29) , for every 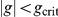 there are at least two equilibria at different values of 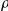. Note that 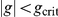 can happen at either sign of 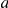, *i.e.* both for attracting and repelling inhomogeneities.

**Figure 7:**
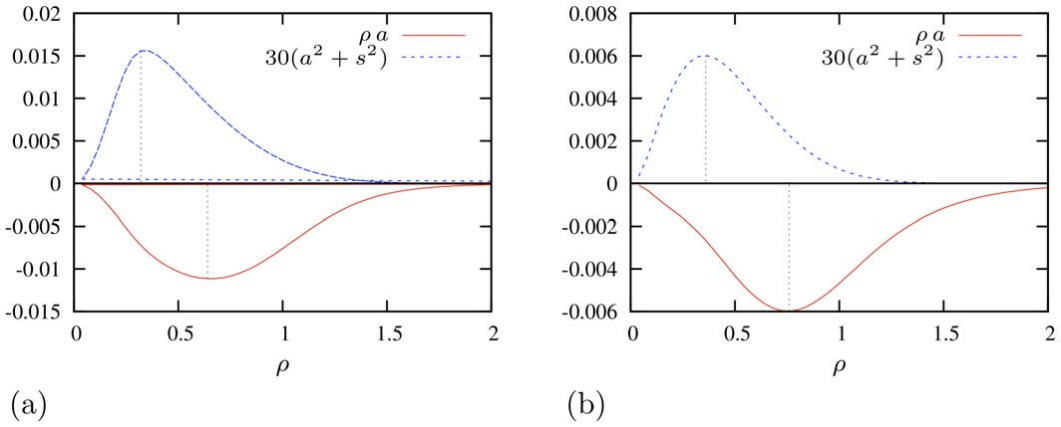
Graphs for graphical solution of stability of “pinning equilibrium”. (a) 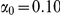, (b) 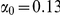. Diffusivity is assumed 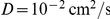. The meanings of the vertical axes are different for different curves and are designated in the legends.

Standard calculations give that an equilibrium at a distance 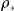 from the inhomogeneity will be stable in linear approximation if and only if the following two conditions are satisfied simultaneously:
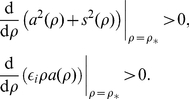
(31)The stability conditions (31) can be easily checked graphically, and the graphs of the two functions involved are shown in figure 7. These conditions require that both functions should be increasing at 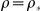. The first inequality does not depend on the sign of 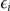, and it therefore demands that 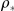 is smaller than the position of the maximum of 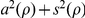 (the blue dashed curves). For the second inequality the situation is more complicated as it depends on the sign of 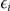. For the case 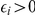, *i.e.* repelling inhomogeneity with excitability higher than 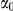, the second stability condition demands that the position of the equilibrium is to the right of the minimum of 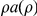, which is shown by the solid red curve. For both values of 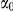 shown in figure 7, and also for all 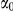 in between, as we have checked, this is incompatible with the first condition, as the red minimum always happens to the right of the blue maximum. For 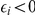, *i.e.* attracting inhomogeneity with excitability lower than 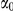 around it, the second stability condition demands that the position of the equilibrium should be to the left of the red minimum, which is a requirement that is weaker than the first condition, as all points to the left of the blue maximum are also to the left of the red minimum. So, in our model there cannot be a stable equilibrium near a repelling inhomogeneity, but only near an attractive inhomogeneity. Intriguingly, if the relative position of the two extrema was different, *i.e.* the red minimum was to the left of the blue maximum, it would create a paradoxical possibility of a stable equilibrium occuring due to interaction with a repelling inhomogeneity. We are not aware of any reasons why this could not happen in some models, but it does not happen in our present model in the range of parameters that we are interested in.

Thus, these theoretical predictions based on the response functions of the vortices suggest that stable pinning of a spiral wave in our model may be to lowered-excitability sites only, while in the experimental and simulations described in [7] the pinning to inhomogeneities of either sign was observed. We have a closer look at this seeming contradiction below.

**Figure 8:**
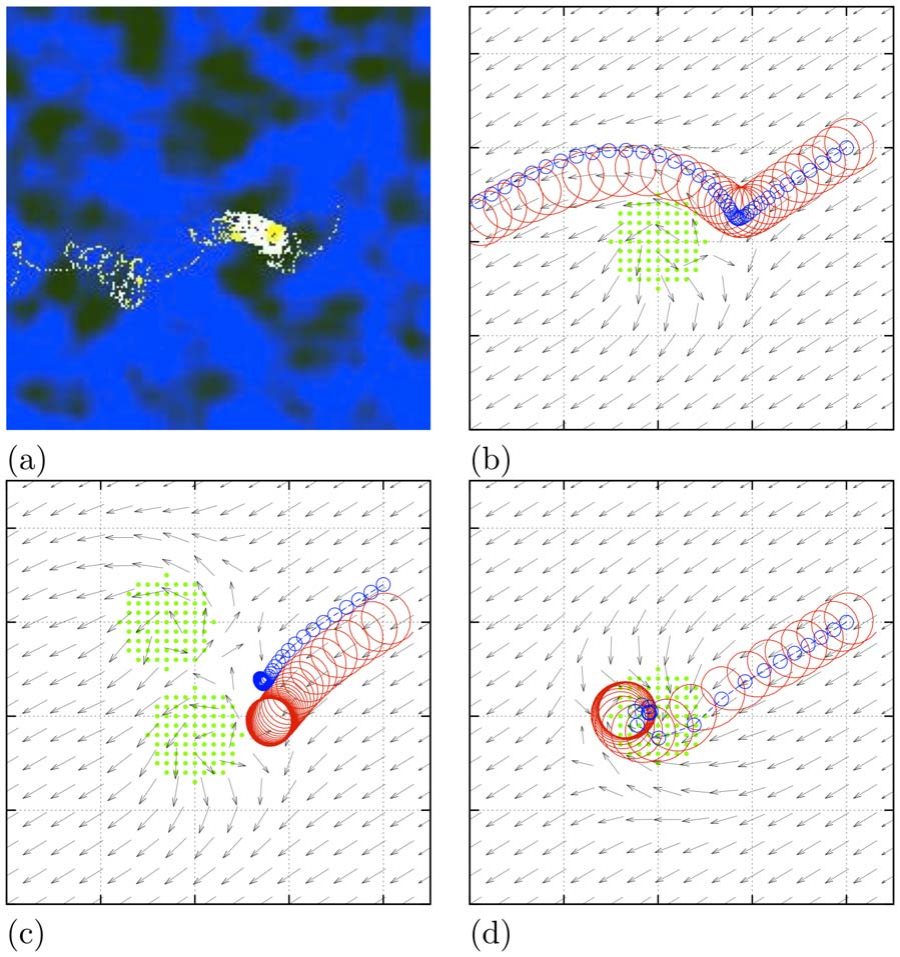
Pinning of spiral wave's drift to localized inhomogeneities. (a) An extension of the drift trajectory shown in figure 8D in [7] with temporary pinning to a high-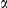 cluster. This is a 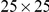-cell fragment of a tip path in a simulation in a box of 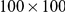 cells, 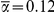, 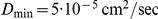, 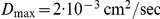, 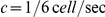. The colour background shows distribution of 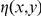, smoothened by sliding averaging, (greenish) dark corresponds to high 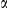 and (blue) light corresponds to low 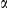. (b) Drift caused by a repelling circular inhomogeneity (green dots show affected cells) of radius of 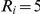 cells. Red solid line is the tip trajectory in a 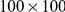-cell simulation, 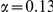 in the bulk of the medium and 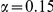 within the disk, and diffusivity 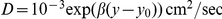, where 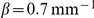 and 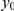 is the middle of the box. The arrows represent the corresponding direction field in the ODE model (27) . The small blue open circles are the instantaneous centres of rotation of the spiral predicted by the ODE model and shown at intervals corresponding to one rotation period of the spiral. These instantaneous centres of rotation of the spiral can be thought of as sliding period-averaged positions of the tip, and make a drift trajectory as predicted by the ODE model. (c) Two repelling inhomogeneities of the same kind as in (b) can stop the drift altogether. (d) Attractive inhomogeneity with lowered excitability, 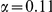, within the disk of the same size as in (b). Now the spiral is permanently stalled behind the heterogeneity. Here and elsewhere, the tip of the numerical spiral at any given moment of time is defined as an intersection of isolines 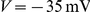 and 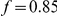 (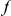 is the dimensionless inactivation gating variable for the slow inward current).

Firstly, the pinning observed in experiments and simulations was not permanent but temporary. An explanation for that could be that the pinning persisted only until the gradient force exceeded the tear-off threshold (30). However, it is also possible that the pinning was temporary because it was really a slow-down near an unstable equilibrium in the vicinity of a repelling inhomogeneity. Panel (a) in figure 8 reproduces the tip trajectory in a “pinning” event from [7], revealing that it was actually only a temporary stall between two fast-drift episodes. Panel (b) illustrates that this sort of stalling is easily reproduced in deliberately arranged simulations and is well described by the ODE model (27).

Secondly, a certain mutual allocation of repelling heterogeneities may cause ‘permanent’ pinning, again until the parameters change. This is illustrated in figure 8 (c). There are two identical repelling inhomogeneities. For the given initial position of the spiral wave, if only the lower inhomogeneity was present, the drift would proceed along a trajectory similar to that in panel (b). However this drift is disallowed by the presence of the upper repelling inhomogeneity, hence the spiral stops at a point of equilibrium of three forces: the constant dragging force and the two repulsion forces from the two localized repelling inhomogeneities.

Panel (d) in figure 8 is given for completeness, to illustrate the more straightforward case of pinning in the vicinity of an attracting inhomogeneity. It is worth noticing a simple phenomenological difference between pinning to a repulsive inhomogeneity and to an attractive one: for the former, the spiral wave stops *in front* of the inhomogeneity, and for the latter, *behind* it.

There are several factors responsible for the quantitative discrepancies seen in figure 8(b–d) between the theoretical predictions for the trajectories of the spiral drift and the trajectories obtained from direct numerical simulations: large value of 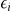 affecting the applicability of the asymptotic theory, the crudeness of the cell structure affecting the behaviour of the direct simulations as compared to the continuous limit, and also the finite 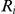 used in simulations as compared to the small-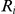 limit assumed in the theory. However, the theoretical trajectories and those obtained from direct numerical simulations are in good qualitative agreement, so the asymptotic theory works really well for this complicated arrangement, despite all the simplifications made.

Naturally, with the random distribution of heterogeneity, as present in the experiments and the numerical simulations of the ischaemic border zone, all of the above scenarios with pinning to inhomogeneities of either sign could take place from time to time. In some experiments the pinning locations subsequently became sources of ectopic waves of excitation and therefore were associated with points of higher excitability. In other experiments, the pinning locations never produced the ectopic waves, which suggested that the pinning inhomogeneity had the lowered excitability. Understanding that there are different mechanisms of pinning to attractive inhomogeneity with lowered excitability and to a (group of) repelling inhomogeneity(s) with elevated excitability provides an explanation for these seemingly contradicting experimental observations.

### Generation of a 3D turbulent pattern

The asymptotic theory of spiral and scroll drift is valid for PDEs, describing continuous media. The theory might not be applicable if the discreteness of the cell structure is significant when the diffusivity is small. Thus our findings here are purely empirical, based on direct numerical simulations. The role of discreteness in 2D dynamics was extensively analysed in [6], [7] so here we concentrate on 3D aspects.

**Figure 9:**
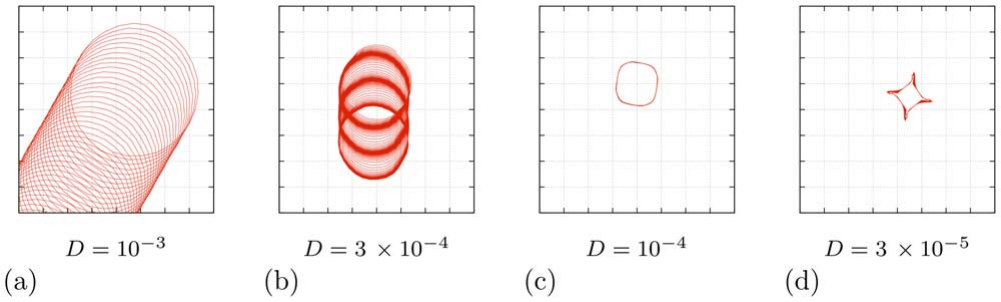
Two-dimensional curvature drift. 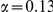
, 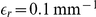. Shown are tip trajectories in system (13) in 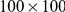-cell box for various 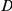, as shown under the panels, in 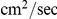. Smaller diffusivity means stronger effect of the discreteness of the tissue, which can stop the drift altogether (the grid of dotted lines designates individual cells).

The effect of dicreteness is to a certain extent similar to that of heterogeneities, *i.e.* it can hinder the drift caused by the smooth parametric or diffusivity gradient. This is illustrated in figure 9, where we present simulations of 2D curvature-induced (electrofophoretic) drift, for different values of diffusivity at 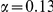 and positive 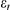. As can be seen from Figure 5 (a), at 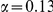 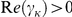, so 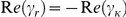 is negative which corresponds to the drift in the negative 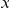 direction in figure 9.

It can be seen that in figure 9 , in line with continuous limit predictions, as diffusivity decreases, so does the spatial scale of the spiral tip trajectory. Further still there is another effect which has an entirely discrete nature: as diffusivity becomes too small, the drift of the spiral stops altogether. Panel (b) indicates that there is a range of diffusivities at which the longitudinal component of the drift (which corresponds to the filament tension 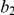 and is smaller in absolute value than the lateral component, see Figure 5 (a)), ‘freezes out’, while the lateral component is still observed, so the drift proceeds along the vertical grid line.

Note that change of filament tension due to relatively small discreteness is a generic feature of excitable media, and has been reported in FitzHugh-Nagumo [23] and Barkley [42] models.

**Figure 10:**
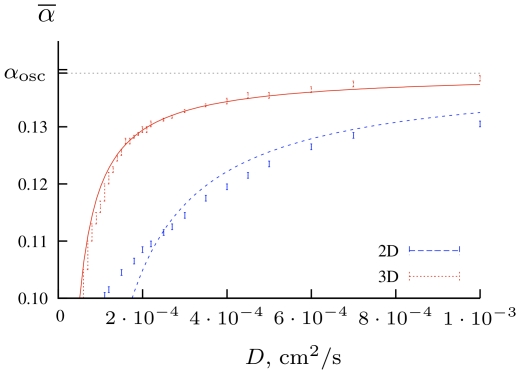
The transition curves obtained in simulations of a 2D and a thin 3D layer of cells. 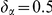
. Below each corresponding curve, the system is quiescent, above the curve and below the 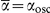 line, focal sources are observed. We also show the best fits, with the weight 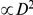, by the theoretical dependence 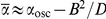 suggested in [6].

Another important effect of the tissue discreteness is due to the role of microscopic heterogeneities of parameter 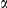, defined by equation (2) , in the generation of ectopic foci and breakup of excitation waves. In presence of the microscopic heterogeneity, 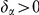, a macroscopically homogeneous tissue, with 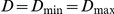, 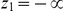 in (3) and (4) , may either show spontaneous focal sources or be quiescent depending on particular combination of 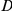, 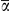 and 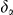. The critical curves in the 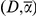 plane, separating the automatic and exctiable regimes (corresponding to the zones III and V in figure 3 and transition between them), are shown in figure 10 , for 2D and 3D cases. We obtained the 3D curve from direct simulations on a thin three-dimensional grid of 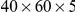 cells at 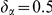. The transition curve obtained from 2D simulations, as in [6], is shown on the same graph for comparison. One can see that position of the 3D transitional curve is elevated compared to the 2D transitional curve. This elevation is due to the fact that every cell in 3D is connected to more neighbours, which increases the load on the automatic cells surrounded by non-automatic environment. Therefore, in 3D it takes more automaticity to overcome the coupling with the quiescent neighbours, so in 3D simulations the same regimes are observed at different values of parameters 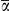 and 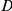 than in 2D simulations.

**Figure 11:**
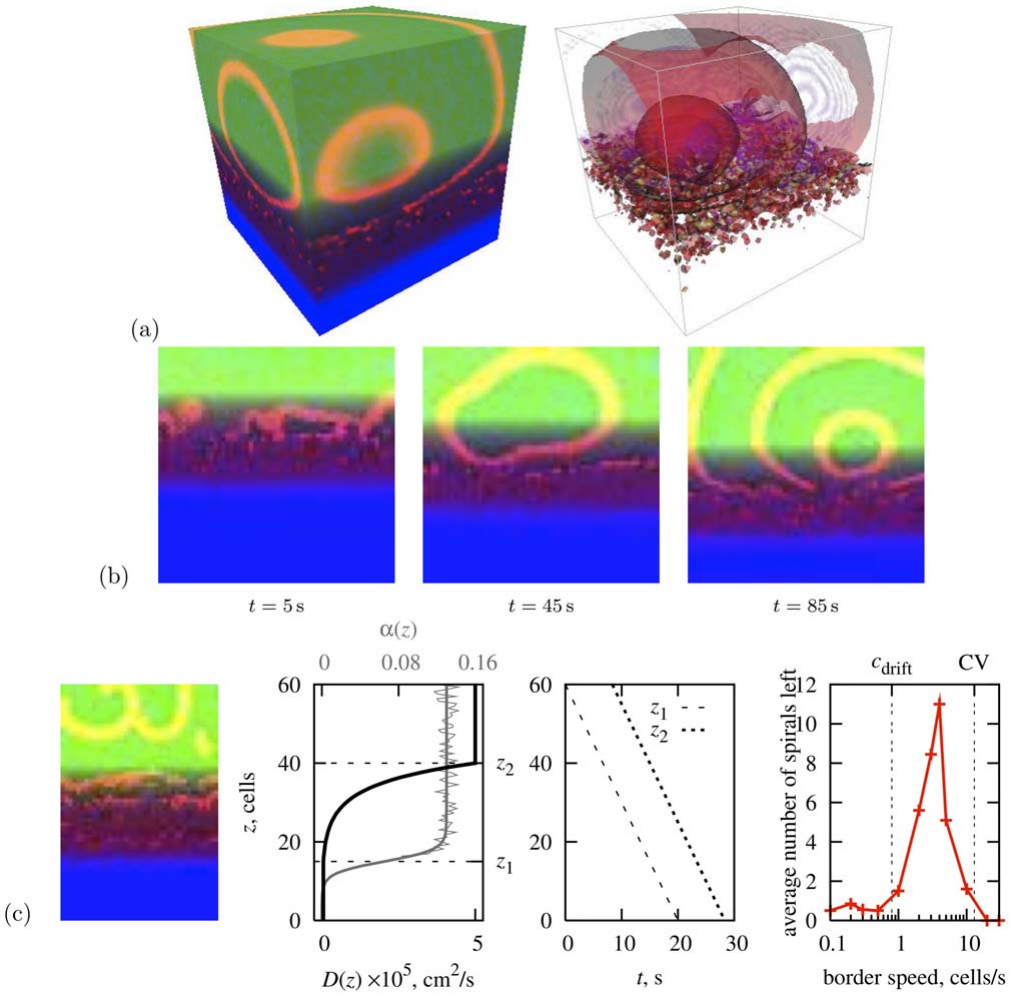
The ischaemic border zone in three dimensions. “Toy” set of parameters: 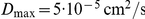, 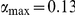, 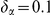. (a,b) Box size 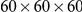 cells, border speed 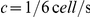. (a) Snapshot of activity on the surface and inside the box (red semi-transparent surfaces are excitation fronts). (b) Activation patterns on a middle cross-section of the box. (c) Schematic of the study of spirals' probability to escape to the well coupled zone: a snapshot through the middle of a thin 3D layer of cells (box size 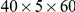, border speed 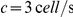); corresponding distribution of 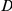, 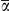 and 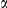; movement of boundaries with time; and average number of spirals left in the box after passing of the border zone, as function of the its speed. Here 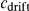 is a typical drift velocity and CV is a typical conduction velocity.

We performed numerical simulations of the 3D tissue slab with diffusivity and excitability profiles shown in figure 11 (c). The lower layer contained fully uncoupled cells with excitability 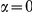. This layer corresponded to the region IV in figure 3 , “a quiescent state where wave propagation is not possible”. To reveal the above described effects of tissue discreteness, we performed simulations using three different sets of parameters 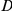, 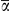 and 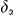 defining properties of the upper layer. We used 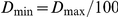 in all cases.

**Figure 12:**
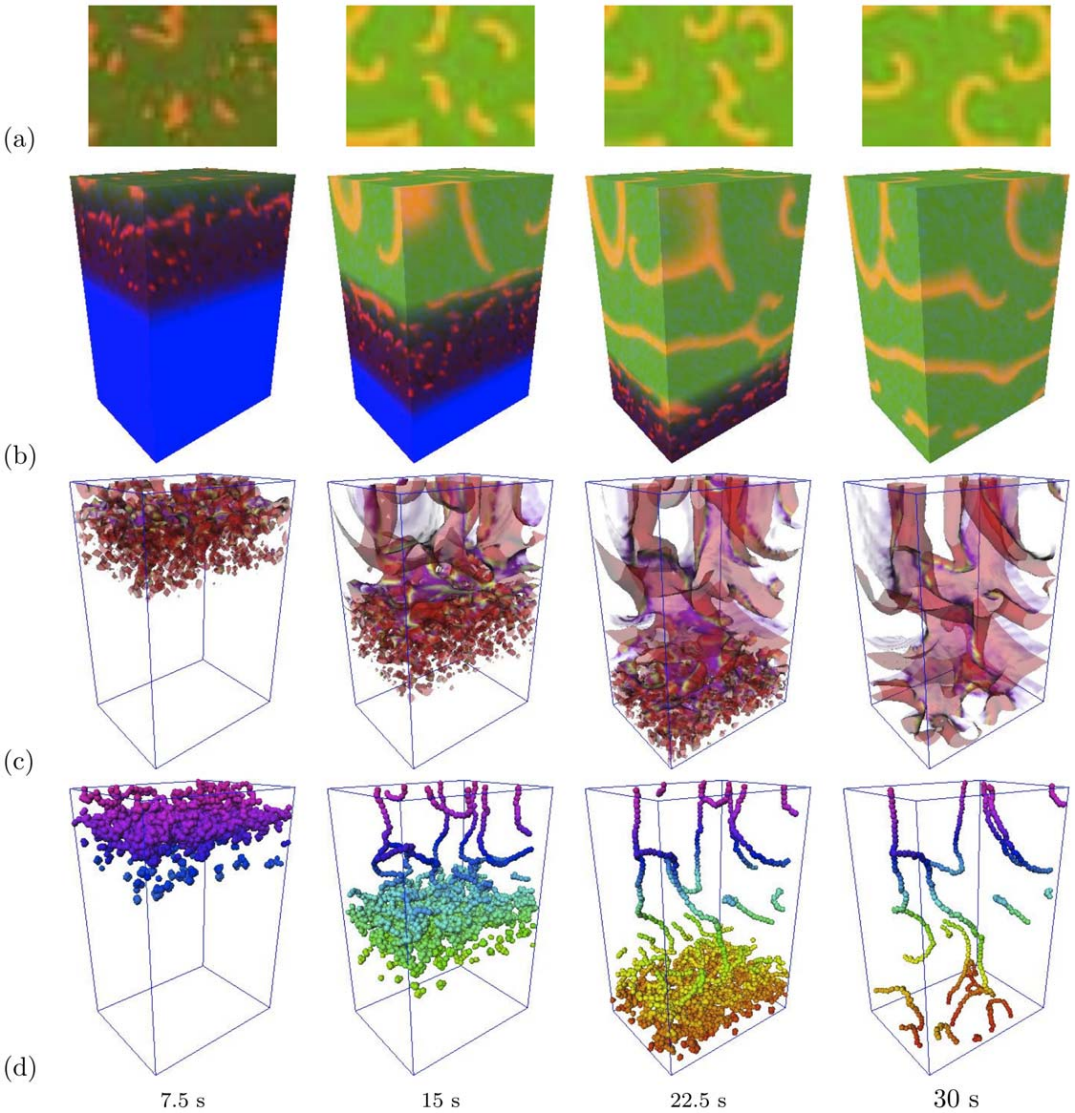
Moving border zone in 3D: vortex formation. “Toy” set of parameters: 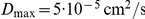, 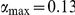, 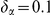, box size 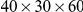 cells, border speed 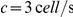. Left to right: successive moments of time. (a) Activation patterns at the top face of the box. (b) 3D view of activation patterns at the surfaces of the box. (c) Excitation fronts as semi-transparent surfaces. (d) Vortex filaments visualized as phase singularities where simultaneously 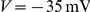 and 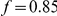. See also the Supplementary Video S1.

The first, “toy” set of parameters (figures 11 and 12) was 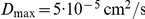, 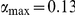, 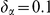. At the small 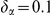 the number of cells getting above 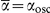 line will be small, resulting in further elevation of the 3D transitional curve compared to the 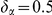 shown in figure 10 . Therefore, the upper layer with this set of parameters 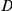, 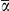 and 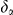 still corresponded to the region V “a quiescent state where wave propagation is possible”, which ensured a transition from what is described as region III “fragmented ectopic waves “ within the middle layer to region V in the top layer. The value 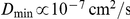 was below the physiologically meaningful range, so simulations with the smaller 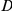 were more of a mathematical excercise, which allowed however, due to the smaller spatial scales involed, to perform a relatively detailed study despite the computational expences of the three-dimensional model. The principal conclusion was then tested with the more physiologically relevant set of paramers.

**Figure 13:**
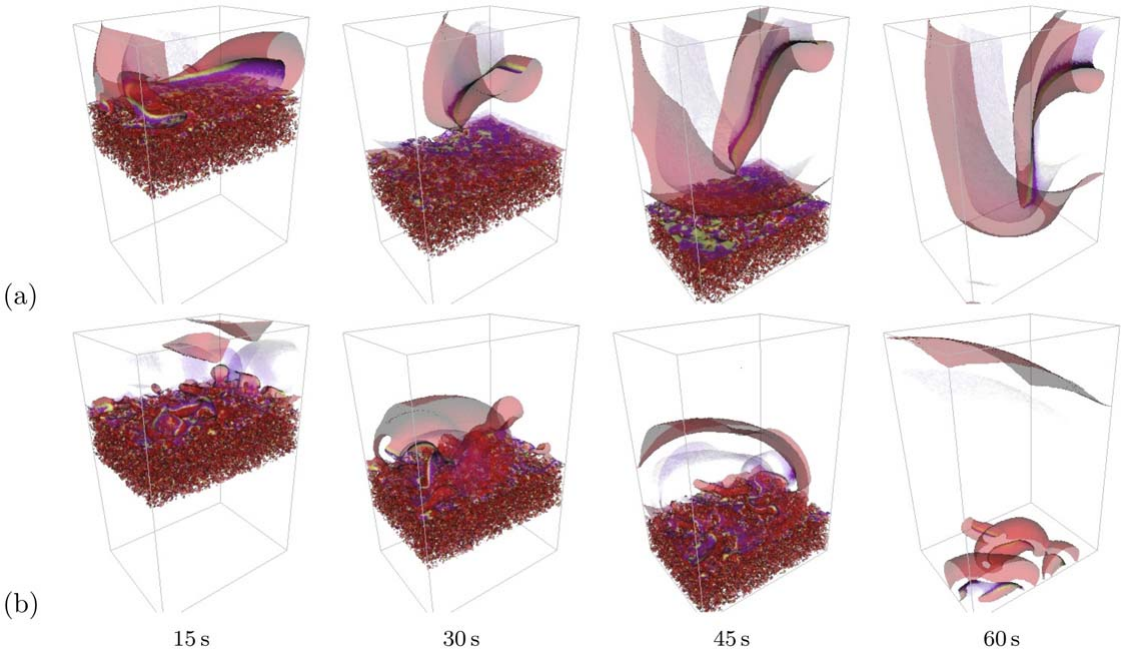
Vortex formation by moving border zone. “More realistic” sets of parameters: 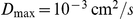, 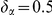, box size 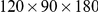 cells, border speed 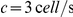. 3D views of activation patterns as in figure 12 (c): (a) 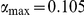; (b) 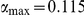. See also the Supplementary Video S2.

The two “more realistic” sets of parameters (figure 13) were 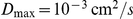, 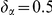, with either 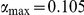 or 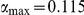, both corresponded to the region V “a quiescent state where wave propagation is possible” (below the critical line in figure 10), which also ensured the transition from region III to region V within the middle layer. This sets of parameters were “more realistic” in terms of the value of diffusivity more relevant to physiologically meaningful range.

Figure 11 presents a simulation with the “toy” set of parameters in a box size 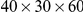 cells and a relatively slow border speed of 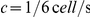. Panel (a) shows small ectopic sources giving rise to multiple ectopic “bubbles” in 3D, also shown in cross-sections from the model's cube of cells in figure 11 (b). The wavefronts from multiple smaller ectopic sources fused into larger wavefronts which were spreading toward the upper, better coupled layers of the border zone. No scroll wave activity is observed in the upper zone. When the transitional border zone has passed down, the cube is left without ectopics and all activity is ceased.

Figure 11 (c) illustrates the probability of the spirals' escape as a function of the speed of the border in a thin 3D grid of cells. When the border moves too slow, it tends to “drag” the spiral waves with it, so none penetrate into the outer zone, as was the case in the simulation shown in panels (a,b). When the border speed is too high, then again no spiral waves are observed in the better coupled upper layer, as they do not have enough time to develop. So, as can be seen from the far right graph in Figure 11 (c), the escapes are possible when the transitional middle layer moves faster than a typical velocity of a spiral wave drift, but slower than the conduction velocity (both speeds are measured for the conditions of suppressed coupling which can be found in the border zone). In particular, the maximal number of spirals was observed at the border speed of 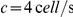. A snapshot half way through a simulation with 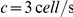, with a few spirals that have already penetrated the outer zone, is shown on the leftmost panel. As we noted earlier, in reality the border zone speed may vary in a very broad range.

Simple escape into the well-coupled upper layer is not enough for the scrolls to cause fibrillation in 3D. Scroll waves are typically born as “scroll rings” with closed filaments. As shown above, in our model, negative filament tension is predicted by the theory for the smaller values of excitability parameter 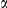. Moreover, the effects of filament tension of either sign can be obstructed by the discrete structure of the model tissue, which is particularly essential in the conditions of the suppressed coupling.

Figure 12 presents a simulation with the “toy” set of parameters in a box size 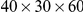 cells and a higher border speed of 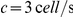, when moving border zone led to generation of multiple scrolls which stayed in the medium after the zone was gone (see also the Supplementary Video S1). Figure 12 (a) shows the top view of the 3D box at four selected instants. To a viewer this will appear as small ectopic sources developing into larger spiral waves. Figure 12 (b) reveals the underlying 3D waves as they would look on the side faces of the box. The 3D scrolls originate deep within the poorly coupled layers of the ischaemic tissue and are spreading upwards as the border zone moves downwards. Figure 12 (c) shows in transparent colors the wavefronts of these newly born scrolls. Finally, figure 12 (d) shows scroll filaments visualized as phase singularities defined as the points where simultaneously 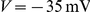 and 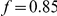. The dense cloud of the singularities corresponds to the area where the microscopic heterogeneities cause multiple wavebreaks. Some of them develop into fully fledged scroll waves, which do not collapse and spread through the whole network of cells to instigate persistent, self-supporting fibrillatory activity. Note that at the value of 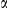 used here, the filament tension is positive, and the scrolls in the upper layer would tend to collapse were it not for the effect of the medium discreteness which according to figure 9 (c,d) is very essential at this artificially low value of 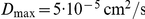. This simulation shown in figure 12 confirms the main conclusion based on the two-dimensional tissue culture experiments and simulations [6], [7]. That is that the key factors of the ischaemic border zone, such as the gradient of coupling strength together with the microscopic heterogeneity and macroscopic gradient of excitability, generate organizing centres of sub-millimeter scale, which then penetrate into the bulk of the well coupled tissue, where the re-entry reaches macroscopic scales.

This main conclusion is supported and reinforced by simulations at larger, more realistic values of 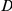, shown in figure 13 (see also the Supplementary Video S2). Stronger coupling results in stronger effective averaging of the microscopic heterogeneities. Hence, for the more realistic 3D similutions, we have increased both the coupling strength 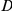 and the microscopic heterogeneity 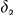. At this bigger 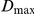 value, the filament tension is already essential, as evidenced by figure 9 (a). We have chosen two values of 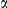 which correspond to a negative and a positive tension of the generated vortex filaments (cf figure 5 (a)). The upper row in figure 13 shows results of simulations with a negative filament tension (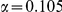). In this simulation, the scroll that penetrated the bulk of the tissue has persisted after the ischemic border zone had disappeared. On the contrast, the lower row in figure 13 shows that for 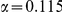, when the scrolls in the upper layer had a positive filament tension, they did not persist, but moved together with the moving border zone. Continuation of the simulation figure 13 (b) led to complete elimination of all activity (not shown). All that is in full agreement with what could be expected from the predictions of the asymptotic theory.

## Discussion

We have considered the quantitative predictions of the asymptotic theory for the forces acting on rotating waves of activity that can form within a recovering ischaemic border. The direct numerical simulations with deliberately arranged conditions confirmed the theoretical predictions for the evolution of the vortices. Now, we can answer the specific questions posed in the Introduction as follows.

1. *“In both experiments and numerical simulations, spiral waves were not static within the border zone. What determines the components of the drift velocity, and why the spiral cores can be dragged together with the moving border zone?”*

- The theoretical analysis of the acting forces shows that regions with suppressed excitability 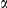 are attracting for spirals, both if applied as a smooth gradient, or as a localized heterogeneity. Conversely, if the upper layer of the boundary layer has a higher excitability, it tends to repel spirals. This implies dragging the cores of the newly born re-entries by the moving transitional border zone down towards the bottom layer with the lowered excitability, and preventing them from escaping into the upper layer and ultimately into the normal tissue with higher excitability.

- At the relatively low values of excitability 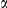 in the upper layer corresponding to 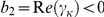, the spirals are repelled from the transitional border layer into the better coupled upper layer with higher diffusivity. This case corresponds to the simulation with 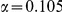 shown in figure 13 (a). In this simulation, the newly born scroll penetrated the bulk of the tissue and persisted even after the recovering border zone ceased to exist.

At the relatively high values of excitability 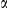 in the upper layer corresponding to 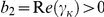, a gradient of diffusivity drives spiral waves towards areas of smaller diffusivity, *i.e.* towards the poor coupled bottom layer. This case corresponds to the simulation with 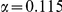 shown in figure 13 (b). In this simulation, the newly born scroll filaments never managed to get far into the upper layer, where the positive filament tension further helped to complete their elimination.

Hence, a relatively high excitability in the upper layer will suppress the transition to fibrillatory-like state for *two* reasons: the gradient of excitability will prevent the cores of spirals or filaments of scrolls from escaping into the more excitable outer zone; and at higher excitability, the gradient of coupling will also drag them away from the better coupled outer zone.

2. *“In both experiments and numerical simulations, the drift of the spirals was interrupted by their “pinning” to clusters of cells. We have shown numerically that these can be cell clusters of either elevated or suppressed excitability. What is the mechanism of such pinning?”*

- The theoretical analysis shows that a combination of acting forces generated by smooth gradients of tissue properties and a localized inhomogeneity in excitability parameter 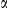 may lead to temporary or permanent pinning of drifting spirals. The chances of pinning depend on the trajectory of the drifting spiral and geometry of the heterogeneity, and it may happen at either sign of the inhomogeneity (*i.e.* locally increased or decreased excitability).

- There is more than one mechanism of pinning. Apart from pinning to an attracting inhomogeneity, the drift can also be stopped by a certain spatial arrangement of repelling inhomogeneities. Even if “permanent” pinning is not achieved, a temporary pinning still may be observed for some finite time if the trajectory of the spiral core passes near an unstable equilibrium. There is also a theoretical possibility of “orbital motion” which however is not realized in the present model at interesting values of parameters.

3. *“In both experiments and numerical simulations, the episodes of spiral drift and pinning alternated. What is the mechanism by which pinning can give way to further drift?”*

- Correspondingly, there is more than one mechanism of unpinning. One is that due to the border zone dynamics, parameters of the tissue may change in such a way that gradient-induced force exceeds the tear-off threshold. The other is that the spiral wave core drifts away from the pinning site because its position there was unstable in the first place.

4. *“One of arrhythmogenic scenarios proposed in* [6], [7] *involved pinning of a spiral wave to a local heterogeneity which persists long enough until the border zone passes and the spiral gets into the better coupled tissue. Is this scenario viable in 3D?”*

- In 3D, in addition to whatever dynamics 2D spiral waves might have, scroll waves exhibit additional dynamics associated with the motion of filament, and characterized by the *filament tension* and the *binormal drift coefficient*. In the considered tissue model, the filament tension is small compared to the binormal drift coefficient, and changes sign in the relevant range of excitability parameter. This means that scroll waves that managed to escape into the well coupled upper zone, might not necessarily immediately collapse.

- The scroll filaments that managed to stay until the tissue is recovered, may not collapse but survive, if the filament tension is negative. These filaments may subsequently generate scroll wave turbulence. Note a nontrivial coincidence following from the asymptotic theory: excitability of the upper layer at the lower range of parameter 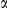 ensures the negative filament tension and hence is a condition of survival of scrolls in that zone, and it also ensures that the specific force caused by the coupling gradient repells the scrolls into the upper, better coupled layer. So here we have a *third* reason a relatively high excitability in the outer layer is “anti-arrhythmic”: at higher excitability, the scrolls in the outer layer are less likely to survive due to 3D effects.

- Further, there are some features revealed by the 2D simulations which are beyond direct applicability of the asymptotic theory. That is the effect of the dicreteness of the medium, which particularly matters at low values of diffusivity. The discretness of the medium can arrest the drift of spiral cores, and when applied to 3D scrolls, the filaments can freeze as long as their curvature is not too high, and the “filament tension” component of their drift freezes sooner than the “lateral binormal drift” component. Therefore, the scroll filaments that managed to stay until the tissue is recovered, may not collapse but survive, as their filament tension is frozen due to low diffusivity. In that case of the “frozen”, zero filament tension, the regime might rather look like a persistent tachycardia similar to the pinned 2D spiral regime.

To summarize, we explored a biophysically plausible mechanism as to how ectopic beats and spreading scrolls of abnormal activity can be generated from the recovering boundary of acutely ischaemic tissue. Complex boundary behaviour in heterogeneous cell network was modeled with certain assumptions and simplifications, extensively discussed in our previous publications [6], [7].

With all the assumptions and limitations, the following combined conclusions can be made based on the *in vitro* and *in silico* data from our previous publications and the current study. First, the data suggested that the combination of the two gradients (*i.e.* the spatial gradient in cell-to-cell coupling and the temporal gradient in excitability/automaticity) ensured that somewhere within the border zone there was a region where multiple ectopic sources were continuously being formed. They were highly localized focal points of activity, with activation spreading only to a few surrounding cells. Number of ectopic sources and specific window of conditions when they occured were affected by the degree of the network heterogeneity. Secondly, the data argued that if the ectopically active layer was sufficiently wide and/or the overall cell automaticity rose, ectopic sources developed into target-like waves. If coupling gradient and automaticity levels remained spatiotemporally fixed, the pattern of target-like sources persisted and no spiral activity was observed. However, when cell automaticity rose and/or border zone moved in space, the propagation patterns became non-stationary. This led to multiple wavebreaks resulting in spiral generation activity. The spiral waves typically demonstrated start-stop drifting behaviour, as a result of competing forces between pinning force due to local heterogeneity and a gradient-induced directional drift. The likelihood of a spiral escape into the better coupled upper tissue zone depend on the speed at which the border zone moves in space.

Our extrapolation of 2D events into 3D is more theoretical, as tissue culture experiments similar to those described in [5], [6] are not feasible in 3D. Still, this extrapolation has shown that the border zone can give rise to 3D analogues of spirals, the scroll waves. If a scroll wave escapes into a better coupled tissue it will not necessarily cause fibrillation, because the scroll wave with positive filament tension have tendency to collapse. However, our simulations have shown that this collapse of newly generated scrolls is not inevitable and, instead, scroll filaments can stabilise or, in case of negative filament tension, expand and multiply leading to a fibrillation-like state.

In this study, we considered the asymptotic theory's quantitative predictions for the forces acting on a cardiac re-entry, and causing its drift, in the vicinity of the ischaemic border zone. The theoretical predictions allow to tell apart and highlight different mechanisms of arrythmogenesis by the ischaemic boder zone in three-dimentiontional settings. The direct numerical simulations with deliberately arranged conditions confirmed the theoretical predictions for the drift.

We fully realize that in vivo, the above considered scenarios will be affected by multiple additional factors. These might include excitability kinetics different from the simplified generic model we used here, presence of highly excitable Purkinje fibers, macroscopic myofiber orientation, coronary vessels, fibrous or fat deposits, transmural differences in myocytes metabolic activity and their sensitivity to ischaemia. Yet, with all its limitations, this study represents one of the first attempts to theoretically explore a very complex set of highly arrhythmogenic conditions that can occur on the boundary of the recovering ischaemic tissue.

## Supporting Information

Appendix S1 Describing details of the Beeler-Reuter-Pumir kinetic model, and of our numerical scheme.
(PDF)

Video S1 Movie illustration to figure 12.
(MPG)

Video S2 Movie illustration to figure 13.
(MOV)
