# Supplementary material for: Evolution of Spiral and Scroll Waves of Excitation in a Mathematical Model of Ischaemic Border Zone
Source: PLoS One. 2011 Sep 15;6(9):e24388. doi: 10.1371/journal.pone.0024388 (PMC3174161; doi:10.1371/journal.pone.0024388)
Supplement: Appendix S1 — Describing details of the Beeler-Reuter-Pumir kinetic model, and of our numerical scheme. (PDF) [file pone.0024388.s001.pdf]

Evolution of spiral and scroll waves of excitation in a mathematical model of ischaemic border zone

V. N. Biktashev[1,*], I. V. Biktasheva[2], N. A. Sarvazyan[3]
1 Department of Mathematical Sciences, University of Liverpool, Liverpool, UK
2 Department of Computer Science, University of Liverpool, Liverpool, UK
3 Pharmacology and Physiology Department, The George Washington University, Washington DC, USA
∗ E-mail: Corresponding vnb@liv.ac.uk

Appendix

Beeler-Reuter-Pumir model

Equations are the same as in the original paper by Beeler and Reuter, with m gate replaced by its quasi-stationary value:

$$\begin{aligned}
\dot{V} &= -(1/C_m)(i_{K_1} + i_{x_1} + i_{Na} + i_s) + I_{ext}, \\
\dot{x_1} &= \alpha_{x_1}(1 - x_1) - \beta_{x_1} x_1, \\
\dot{h} &= \alpha_h(1 - h) - \beta_h h, \\
\dot{j} &= \alpha_j(1 - j) - \beta_j j, \\
\dot{d} &= \alpha_d(1 - d) - \beta_d d, \\
\dot{f} &= \alpha_f(1 - f) - \beta_f f, \\
\dot{[\mathrm{Ca}]}_i &= -10^{-7} i_s + 0.07(10^{-7} - [\mathrm{Ca}]_i),
\end{aligned}$$

where

$$\begin{aligned}
m &= \alpha_m/(\alpha_m + \beta_m), \\
i_{K_1} &= 0.35(0.3 - \alpha)\left[\frac{4\,\mathrm{e}^{0.04(V+85)} - 1}{\mathrm{e}^{0.08(V+53)} + \mathrm{e}^{0.04(V+53)}} + \frac{0.2(V+23)}{1 - \mathrm{e}^{-0.04(V+23)}}\right], \\
i_{x_1} &= g_{x_1} x_1, \\
i_{Na} &= (g_{Na} m^3\, h\, j + g_{Na,c})(V - E_{Na}), \\
i_s &= g_s\, d\, f(V - E_s), \\
\alpha_{x_1} &= \frac{0.0005\,\mathrm{e}^{0.083(V+50)}}{\mathrm{e}^{0.057(V+50)} + 1}, \\
\beta_{x_1} &= \frac{0.0013\,\mathrm{e}^{-0.06(V+20)}}{\mathrm{e}^{-0.04(V+20)} + 1}, \\
\alpha_m &= \frac{V + 47}{1 - \mathrm{e}^{-0.1(V+47)}}, \\
\beta_m &= 40\,\mathrm{e}^{-0.056(V+72)}, \\
\alpha_h &= 0.126\,\mathrm{e}^{-0.25(V+77)}, \\
\beta_h &= \frac{1.7}{\mathrm{e}^{-0.082(V+22.5)} + 1}, \\
\alpha_j &= \frac{0.055\,\mathrm{e}^{-0.25(V+78)}}{\mathrm{e}^{-0.2(V+78)} + 1}, \\
\beta_j &= \frac{0.3}{\mathrm{e}^{-0.1(V+32)} + 1},
\end{aligned}$$

$$
\begin{aligned}
\alpha_d &= \frac{0.095\,\mathrm{e}^{-0.01(V-5)}}{\mathrm{e}^{-0.072(V-5)}+1}, \\
\beta_d &= \frac{0.07\,\mathrm{e}^{-0.017(V+44)}}{\mathrm{e}^{0.05(V+44)}+1}, \\
\alpha_f &= \frac{0.012\,\mathrm{e}^{-0.008(V+28)}}{\mathrm{e}^{0.15(V+28)}+1}, \\
\beta_f &= \frac{0.0065\,\mathrm{e}^{-0.02(V+30)}}{\mathrm{e}^{-0.2(V+30)}+1}, \\
g_{x_1} &= 0.8\frac{\mathrm{e}^{0.04(V+77)}-1}{\mathrm{e}^{0.04(V+35)}}, \\
E_s &= -82.3 - 13.0287\log([\mathrm{Ca}]_i), \\
I_{ext} &= \nabla\left(D\nabla V\right).
\end{aligned}
$$

Most of the parameters of the model were fixed in all simulations, at the following values: $C_m = 1$, $g_{Na} = 2.4$, $g_{Na,c} = 0.003$, $E_{Na} = 50$, $g_s = 0.045$. Parameters D and α were varied in space and time, as explained in the text.

Numerical methods

Spatial approximation uses regular cuboidal grid with space step $\Delta x = 0.03\,\mathrm{mm}$ so that one grid node represents one tissue cell, and the cells are connected to each other by Ohmic contacts (bidomain effects are not thought to be important for the phenomena under consideration). Hence the Laplacian term in three spatial dimensions is

$$
\nabla\left(D\nabla V\right) = \sum_{j=1}^{3}\frac{\partial}{\partial x_j}\left(D(x_1,x_2,x_3)\frac{\mathrm{d}V}{\mathrm{d}x_j}\right)
$$

where e.g. for $x_1 = x$ we implement a conservative scheme

$$
\begin{aligned}
&\frac{\partial}{\partial x}\left(D(x,y,z)\frac{\mathrm{d}V}{\mathrm{d}x}\right) \\
\approx\ &\frac{1}{\Delta x^2}\left[\frac{D(x+\Delta x,y,z)+D(x,y,z)}{2}(V(x+\Delta x,y,z)-V(x,y,z))\right. \\
&\left.+\frac{D(x-\Delta x,y,z)+D(x,y,z)}{2}(V(x-\Delta x,y,z)-V(x,y,z))\right]
\end{aligned}
$$

and similarly for $x_2 = y$ and $x_3 = z$. The boundary conditions are non-flux, implemented by setting a 1-cell layer of fictitious nodes around the box, in which the V value was assumed identical to that in the nearest real grid node.

Time stepping is done using fully explicit Euler scheme with a time step of $\Delta t = 0.1\,\mathrm{ms}$.

Gaussian distribution for $\eta(x,y,z)$ is implemented using the Box-Muller 1958 transformation.
